# An assessment of total antioxidant and oxidant parameters and their correlation with embryo quality in in-vitro fertilization patients

**DOI:** 10.61622/rbgo/2025rbgo22

**Published:** 2025-04-30

**Authors:** Utkucan Okuducu, Gökhan Bayhan, Dilek Ulusoy Karatopuk

**Affiliations:** 1 Yüreğir State Hospital Department of Gynecology Adana Turkey Yüreğir State Hospital, Department of Gynecology, Adana, Turkey.; 2 Süleyman Demirel University Faculty of Medicine Hospital Department of Gynecology Isparta Turkey Department of Gynecology, Süleyman Demirel University Faculty of Medicine Hospital, Isparta, Turkey.; 3 Süleyman Demirel University Faculty of Medicine Hospital IVF Center Isparta Turkey Süleyman Demirel University Faculty of Medicine Hospital, IVF Center, Isparta, Turkey.

**Keywords:** Infertility, Fertilization in vitro, Oocytes, Follicular fluid, Antioxidants, Oxidants, Oxidative stress

## Abstract

**Objective:**

In vitro, fertilization is the primary treatment method for infertility. Follicular fluid analysis is an approach used to optimize the results of assisted reproductive techniques. Oxidative stress represents the imbalance between the production of reactive oxygen species and their detoxification. Total Antioxidant and Oxidant Status, and Oxidative Stress Index levels are the main oxidative stress markers. This study investigated the effects of oxidative stress markers on infertility etiology, embryo quality, and success of In vitro fertilization.

**Methods:**

Before enrolling in the ICSI-ET cycle, participants had their FSH and LH levels assessed on the second day of the cycle. The ovarian degrees of the participants were evaluated by transvaginal ultrasonography. Participants underwent controlled ovarian stimulation using the GnRH antagonist protocol. TV-USG and serial E2 measurements were performed at appropriate intervals to follow follicular development. Follicle sizes, quantity, and endometrial thickness were recorded. Total Antioxidant and Oxidant Status, and Oxidative analyses were conducted using Rel Assay Diagnostics Assay Kits.

**Results:**

The average number of total oocytes in the participants was 10.25±6.66, and the average of mature M2 stage oocytes was 6.71±3.72. The average number of fertilized oocytes was 4.65±2.81. Fertilization rates were calculated as approximately 54.75±25.58%. A statistically significant positive correlation was found between embryo quality and serum Total Antioxidant Status levels (p=0.004). Similarly, a significant positive correlation was observed between embryo quality and follicular Total Antioxidant Status values (r = 0.42, p = 0.01).

**Conclusion:**

This study concluded that oxidative stress markers affect certain stages of the IVF treatment process.

## Introduction

Defined as the inability to conceive after a year of regular, unprotected intercourse or the inability to carry a pregnancy to term, female infertility arises from a myriad of etiological factors encompassing physiological, anatomical, endocrine, and environmental determinants.^(1)^ Treatment strategies for female infertility are tailored to address underlying etiologies, ranging from lifestyle modifications and pharmacological interventions to surgical procedures and assisted reproductive technologies, including in vitro fertilization (IVF).^(2)^

In vitro fertilization stands at the forefront of assisted reproductive technologies, offering a beacon of hope to millions of individuals grappling with infertility. Today, IVF is a mainstream treatment modality for various etiologies of infertility.^(3)^

Follicular fluid (FF) is a specialized fluid milieu found within ovarian follicles, which plays a pivotal role in the maturation and development of oocytes during folliculogenesis. It creates an optimal microenvironment for oocyte growth and maturation.^(4)^ FF is produced by the granulosa cells that surround the developing oocytes. Furthermore, FF serves as a reservoir of biomarkers that can provide valuable insights into ovarian physiology, follicular development, and reproductive health. Analysis of FF components has emerged as a non-invasive approach for assessing ovarian function, predicting oocyte quality, and optimizing outcomes of assisted reproductive techniques.^(5)^

Oxidative stress represents an imbalance between the production of reactive oxygen species and their detoxification. It plays a pivotal role in the pathogenesis of numerous chronic diseases. Total Antioxidant Status (TAS) is a critical parameter in oxidative stress research. TAS examines the cumulative effect of antioxidants in biological fluids, foods, and dietary supplements.^(6)^ It encompasses a broad spectrum of antioxidants, including enzymatic and non-enzymatic components. TAS is an exemplary marker for analyzing the body's anti-oxidative defense mechanisms. Its significance is paramount in understanding the etiology of diseases associated with oxidative stress. Fertilization and the subsequent battle against oxidative stress underscore the significance of TAS.^(7)^

This study examined the impact of TAS, Total Oxidant Status (TOS), and Oxidative Stress Index (OSI) levels in the serum and FF of infertile patients on the etiology of infertility, embryo quality, and the success of IVF. It scrutinized the feasibility of employing these parameters as predictive markers for the success of IVF treatment.

## Methods

The research was conducted at the IVF unit of the Department of Obstetrics and Gynecology of a Medical Faculty in Turkey between February 2017 and February 2018. The study included 35 infertile patients, aged between 20 to 44 years, who presented for treatment due to low ovarian reserve (n=6), tubal factor (n=5), male factor (n=6), and unexplained infertility reasons (n=18), and were planned for an Intracytoplasmic Sperm Injection-Embryo Transfer (ICSI-ET) cycle. These patients were devoid of any systemic diseases.

The sample size of the study was calculated using the G*Power 3.1.9.6 (Frans Foul, Universitat Kiel, Germany) program. The sample size was calculated as 32.

Our study is prospective and single-centered. The characteristics of the patients, including age, weight, height, and the presence of any comorbidities, have been meticulously recorded. The Body Mass Index was calculated using the formula (kg/m²). The semen samples obtained from the spouses of the participants, following a sexual abstinence period of 3-5 days and acquired through the method of masturbation, have been subjected to thorough analysis.

Before their enrollment in the ICSI-ET cycle, participants underwent an evaluation of FSH and LH levels on the second day of the cycle. Additionally, TSH and AMH levels were analyzed on a randomly selected day. The patency of the fallopian tubes was assessed between the 7th and 10th days of the cycle through HSG. The ovarian grades of the participants were evaluated on the second day of the cycle via transvaginal ultrasonography (TV-USG) (Voluson 730 Expert vaginal prob. 5-9 MHz).

Patients whose spermogram, ovulation, hysterosalpingography (HSG), ovarian reserve tests, and laparoscopy examinations are found to be within normal parameters are classified as suffering from unexplained infertility.^(8)^ The characteristics of patients within this group:^(9)^

- Infertility persisting for a minimum duration of one year;
- Regular menstrual cycles ranging between 21 to 35 days;
- Evidence of ovulation (mid-luteal serum progesterone >5 ng/ml);
- FSH levels <10 IU/L on the second day of the cycle;
- Regular tubal pattern, as demonstrated by HSG;
- Normal sperm parameters.

In cases where female infertility has been ruled out, and male infertility is attributed to various factors such as hypothalamic-pituitary, testicular, or post-testicular issues, semen analysis outcomes are evaluated according to the World Health Organization (WHO) 2010 criteria as oligospermia, azoospermia, teratozoospermia, asthenospermia, or oligo asthenospermia, or in cases where sperm is obtained through Testicular Sperm Extraction (TESE) for Intracytoplasmic Sperm Injection (ICSI) procedures, these are classified under male factor infertility.^(10)^

Participants with abnormal ovarian reserve test results are categorized as having Diminished Ovarian Reserve (DOR). For the diagnosis of DOR, anti-müllerian hormone, follicle-stimulating hormone and estradiol levels were measured by blood tests. Patients presenting with male factor infertility, tubal pathologies, uterine factors, smoking or endometriosis are excluded from this group.^(11)^

Participants were subjected to controlled ovarian stimulation utilizing the GnRH antagonist protocol. Accordingly:

- On the second day of the cycle, TV-USG was conducted to initiate ovarian stimulation with recombinant FSH (Schering-Plough, Turkey) and urinary FSH (Menogon 75 IU; Ferring) treatment;
- The dosage of gonadotropins was adjusted based on the patient's BMI and age values, responses to previous IVF treatments, AMH levels, and ovarian grading determined through TV-USG;
- For pituitary suppression, when the follicular size reached 13-14 mm or the E2 level was 800 pg/ml, a subcutaneous injection of 0.25 mg/day of Cetrorelix (Merck Serono, Turkey) was administered;
- This regimen was continued until the day of hCG administration.

**Figure 1:**
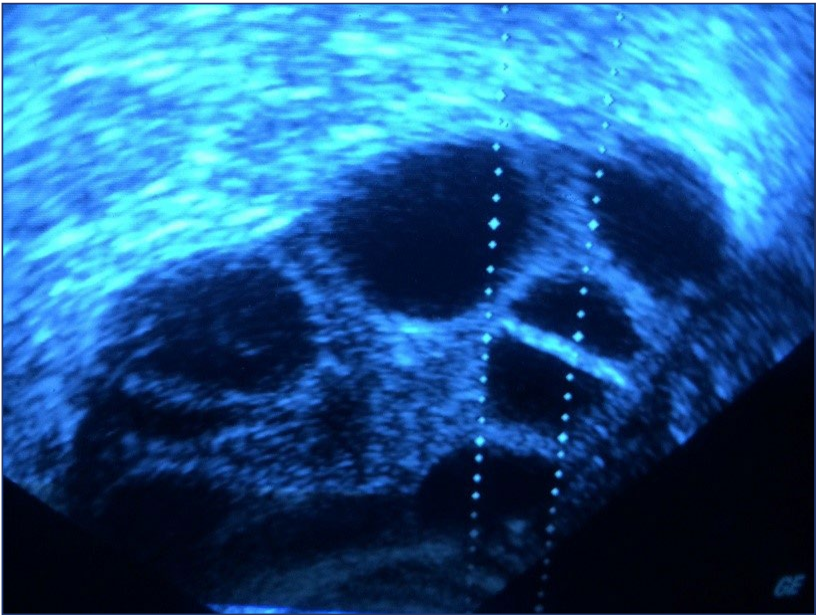
View of the ovary with TV-USG during the OPU procedure

To monitor follicular development, TV-USG, and serial E2 measurements were conducted at appropriate intervals (Figure 1). At each examination, the dimensions, quantity of follicles, and endometrial thicknesses were meticulously documented in the patient tracking forms. When two or more follicles exceeded 17 mm, ovulation was induced with recombinant hCG (subcutaneous injection of 250 µg rHCG). Approximately 36 hours post-hCG administration, participants underwent OPU under general anesthesia. The OPU procedure was executed under general anesthesia, with the patient positioned in dorsolithotomy. A speculum was inserted into the vagina, which was then irrigated with a sterile saline solution. Guided by TV-USG, oocytes were aspirated transvaginally using a double-lumen oocyte aspiration needle of 17 mm in size. The developmental stages of oocytes were documented by embryologists under the light microscope. The quantities of collected oocytes and the number of oocytes in the metaphase stage or those that were fertilized were also recorded.

During the OPU procedure, the embryologist segregated the oocytes extracted from the aspirated FF samples. The residual fluids were centrifuged at 3000 rpm for 10 minutes, following which the supernatant was decanted, and the yellow, translucent FF was transferred into tubes. Venous blood samples were obtained from the participants after a 12-hour fast on the day the OPU procedure was scheduled. These samples were centrifuged at 3000 rpm for 10 minutes to procure serum. Both serum and FF specimens were preserved at -80°C until TAS and TOS levels were determined.

**Figure 2:**
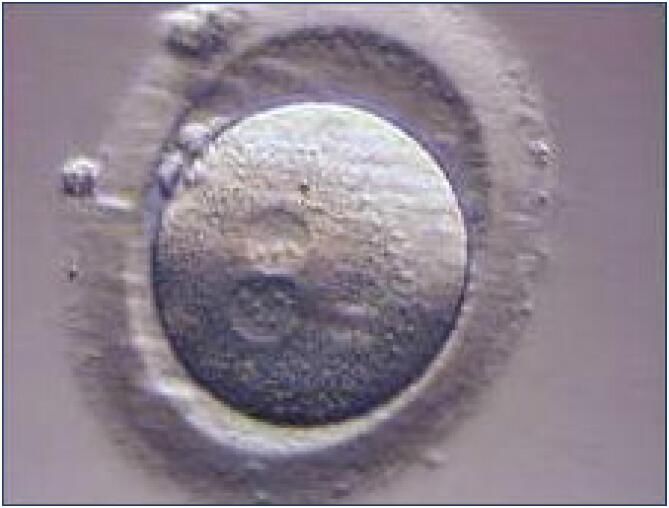
Fertilized oocyte containing two polar bodies

In the studied cohort, the post-liquefaction sperm count and motility derived from the participants’ spouses were meticulously analyzed. The sperms were subjected to swim-up or gradient methods and purified using a modified human tubal fluid medium (PureSperm 40/80, Nidacon International, Mölndal, Sweden). Oocytes were incubated at 37°C in an atmosphere containing 5% CO2, utilizing a modified human tubal fluid medium enhanced with 7% synthetic serum (Vitrolife G-TL, Sweden). The retrieved MII oocytes were placed within Petri dishes containing 5 µl drops of HEPES-buffered solution, upon which the ICSI procedure was executed. Fertilization was assessed one day following the ICSI procedure, with oocytes displaying two pronuclei deemed fertilized (Figure 2).

Embryo evaluation transpired on days 1, 3, and 5 post-ICSI, classifying them into reasonable, fair, and poor quality categories. Initial day assessments were conducted 16-18 hours post-ICSI, interpreting the presence of two pronuclei as indicative of successful fertilization. On day three, cleavage was examined. Embryo quality was determined based on the following:^(12)^

- Good quality: embryos with eight cells, less than 10% fragmentation, and homogeneously sized blastomeres;
- Fair quality: embryos possessing 4-8 cells, 10-25% fragmentation, and minor variations in blastomere sizes;
- Poor quality: embryos with fewer than four cells, over 25% fragmentation, and significant discrepancies in blastomere sizes.

Embryos were further evaluated by day five based on developmental stages, inner cell mass (ICM), and trophectoderm cells. Based on their developmental progression, blastocysts were scored as early, full, and expanding/hatching.^(12)^

- The ICM quality was categorized as good for tightly compacted, numerous cells, fair for easily distinguishable, loosely compacted cells, and poor for scarcely discernible, minimal cell compositions;
- Trophectoderm assessment deemed embryos with numerous cohesive epithelial layers as good quality, those with few loose epithelial layers as fair, and those with very few cells as poor quality.

Embryo transfers were conducted under transvaginal ultrasonography guidance using a Wallace catheter (Sure View, Wallace Ltd., Smiths Medical, Mexico), deciding on transferring one or two embryos based on the patient's age and the quantity and quality of available embryos. All patients were administered intravaginal progesterone gel (Crinone^®^ 8% gel, Merck Serono) starting the day after OPU to facilitate luteal support. The presence of hCG in the blood 12 days post-embryo transfer was interpreted as implantation. In contrast, observing a gestational sac within the uterine cavity via transvaginal ultrasonography five weeks post-transfer was considered a clinical pregnancy. Viability defined as the delivery of a live infant weighing over 500 grams or born beyond the 24th week of gestation—was distinguished from early pregnancy loss, which was categorized as any gestation terminating before the 20th week or with a fetal weight below 500 grams.

TAS and TOS analyses were conducted using Rel Assay Diagnostics Assay Kits via a spectrophotometric method in the samples. The Beckman Coulter AU 5800 autoanalyzer was used for the analyses.

Antioxidant substances in the sample reduced the ABTS radical solution, one of the kit's reactants, to a colorless form. The change in absorbance at 660 nm was measured. This absorbance change is directly proportional to the TAS level in the sample. The method has been calibrated with a stable antioxidant standard solution, Trolox Equivalent (an analog of Vitamin E).^(13)^

Oxidant substances in the sample oxidized the ferrous ion chelator complex, converting it to the ferric ion form. This ferric ion formed a colored complex with the chromogen in an acidic environment. The formation of color is directly proportional to the quantity of oxidant molecules in the sample. This was measured spectrophotometrically, and the TOS value was obtained. The method was calibrated with hydrogen peroxide.^(13)^

Using the obtained TAS and TOS values, the OSI values were calculated as follows. OSI(arbitrary unit)=TOS(μmol H2O2Eq/L)/TAS (μmol Trolox Eq/L)×100 .

- Deep freezer: Kirsch Bosch -80°C. Willstätt-Sand.
- Centrifuge device: Nüve NF 1200 R model refrigerated. Ankara-Turkey.
- Centrifuge device: Eppendorf 5415 R eppendorf. Hamburg-Germany.
- Automatic pipettes: Eppendorf (Germany), Gilson. Hamburg-Germany.
- BECKMAN Coulter: AU5800 biochemistry autoanalyzer. Istanbul-Turkey.
- Vortex device: Nüve NM 100. Ankara-Turkey.
- TAS, Rel Assay Diagnostics, Gaziantep-Turkey.
- TOS. Rel Assay Diagnostics, Gaziantep-Turkey.
- Antioxidant and oxidant capacities may be influenced by chronic systemic diseases.
- Endocrinopathy (hypo/hyperthyroidism, hyperprolactinemia, etc.).
- Endometriosis.
- Autoimmune disease.
- Malabsorption.
- Smoking.

Our study has been designed following the principles of the Helsinki Declaration. The requisite ethical committee approvals have been obtained from the institution where the research was conducted. Written and oral consent was also acquired from the participants of the study prior to their involvement. Voluntary participation has been the foundational principle for inclusion in the study.

The data were analyzed using the SPSS 15.0 statistical software package. Descriptive statistics, namely mean and standard deviation values were reported. The conformity of continuous variables to the normal distribution was assessed via the Kolmogorov–Smirnov test. Continuous variables that adhered to the normal distribution were examined using the Student's t-test, whereas those that did not conform were analyzed with the Mann-Whitney U test. Chi-square and ANOVA tests were employed to compare categorical data. Correlation analysis evaluated the relationship between parameters investigated in serum and FF. A p-value of <0.05 was considered the threshold for statistical significance.

## Results

Table 1 delineates the characteristics of the participants. With the exception of FSH levels (p=0,028), no statistically significant differences were discerned amongst the groups in terms of these particular attributes (Table 1).

**Table 1:** Some characteristics of the participants

	Tubal factor (n=5)	Unexplained (n=18)	DOR (n=6)	Male factor (n=6)	p-value
Age*	26.60±5.45	30.72±3.99	30.55±3.88	27.00±4.19	0.12
BMI* (kg/m^2^)	24.42±2.00	26.91±4.96	27.36±3.60	25.65±4.93	0.65
FSH* (mIU/mL)	7.89±3.39	6.72±2.41	11.17±4.69	6.92±2.14	0.028
LH* (mIU/mL)	6.85±5.13	4.68±2.49	6.22±1.57	6.89±5.48	0.66
AMH* (ng/ml)	3.14±1.91	4.17±3.20	1.73±2.37	3.82±2.49	0.27
TSH* (uIU/mL)	1.62±0.72	2.06±1.24	2.53±1.46	1.63±0.51	0.6

DOR: Diminished Ovarian Reserve;

* Mean±SD

In the study, participants’ serum samples were analyzed for TAS, TOS, and OSI and calculated based on these values to investigate the etiological correlation with infertility. The mean TAS, TOS, and OSI values were found to be 1.37±0.59 μmol Trolox Eq/L, 21.07±11.00 μmol H2O2 Eq/L, and 1960.89±1238.83 arbitrary units, respectively. Statistical analysis revealed no significant differences between groups in terms of TAS, TOS, and OSI values (p>0.05) (Table 2).

**Table 2:** The serum levels of TAS, TOS, and OSI based on the causes of infertility

	General (n=35)	Tubal factor (n=5)	Unexplained (n=18)	DOR (n=6)	Male factor (n=6)	p-value
TAS	1,37±0,59	1,46±0,47	1,27±0,51	1,24±0,48	1,74±0,92	0,38
TOS	21,07±11,0	16,83±7,04	23,63±12,36	21,21±7,30	16,79±12,12	0,47
OSİ	1960,89±1238,83	2205,72±1831,16	2077,66±1195,07	1982,61±941,79	1384,83±1225,89	0,66

TAS: Total Antioxidant Status; TOS: Total Antioxidant Status; OSI: Oxidative Stress Index; DOR: Diminished Ovarian Reserve

The TAS, TOS, and OSI values in the participants’ FF were examined with respect to the causes of infertility. The average TAS, TOS, and OSI values for all participants were respectively 1.77±0.37 μmol Trolox Eq/L, 8.68±4.10 μmol H2O2Eq/L, and 509.31±233.88 arbitrary units. In the analyses, no statistically significant differences were observed in the TAS, TOS, and OSI values among the groups (p>0.05) (Table 3).

**Table 3:** The FF levels of TAS, TOS, and OSI based on the causes of infertility

	General (n=35)	Tubal factor (n=5)	Unexplained (n=18)	DOR (n=6)	Male factor (n=6)	p-value
TAS	1,77±0,37	1,69±0,16	1,76±0,31	1,72±0,42	1,93±0,60	0,69
TOS	8,68±4,10	7,37±3,01	9,85±4,51	9,25±3,42	5,73±2,99	0,15
OSİ	509,31±233,88	452,00±222,57	571,42±273,46	530,73±169,05	343,33±231,88	0,22

TAS: Total Antioxidant Status; TOS: Total Antioxidant Status; OSI: Oxidative Stress Index; DOR: Diminished Ovarian Reserve

Participants’ total number of oocytes averaged 10.25±6.66, with mature M2 stage oocytes averaging 6.71±3.72. The number of oocytes that underwent fertilization averaged 4.65±2.81. Fertilization rates were calculated to be approximately 54.75%±25.58. Furthermore, the ratio of high-quality embryos to the total number of embryos was 25/35. No statistically significant differences between the groups regarding variables related to oocytes and embryos were identified (Table 4).

**Table 4:** Oocytes and embryos of patients according to the causes of infertility

	General (n=35)	Tubal factor (n=5)	Unexplained (n=18)	DOR (n=6)	Male factor (n=6)	p-value
Total oocytes	10,25 ±6,66	11 ±5,61	10,88±7,49	6,46±5,03	11,83 ±5,91	0,43
M2 oocytes	6,71±3,72	6,60±2,40	6,94±3,74	4,50±3,39	8,33±4,03	0,33
Fertilized oocytes	4,65±2,81	4,20±1,30	5,16±3,05	3,00±1,67	5,16±3,65	0,40
Fertilization rate	54,75±25,58	49,81±32,23	54,32±23,16	67,69±29,67	47,15±24,87	0,54
High-quality embryo rate	25/35	3/5	14/18	4/6	4/6	0,89

DOR: Diminished Ovarian Reserve

The study investigated the potential correlation between participants’ TAS, TOS, and OSI values and the quality of oocytes and embryos. The results revealed a statistically significant positive correlation between embryo quality and serum TAS levels (r=0.57, p=0.004). Similarly, a significant positive correlation was observed between embryo quality and follicular TAS values (r=0.42, p=0.01) (Table 5).

**Table 5:** Correlation between TAS, TOS, and OSI and the quality of oocytes and embryos

		Total oocytes	M2 oocytes	Fertilized oocytes	Fertilization rate %	Embriyo grade
Serum TAS	r	0,13	-0,04	0,01	0,58	0,57*
	p	0,94	0,98	0,98	0,74	0,004*
Serum TOS	r	0,23	0,17	0,26	-0,04	0,25
	p	0,17	0,32	0,12	0,79	0,14
Serum OSİ	r	0,09	0,09	0,25	0,10	-0,01
	p	0,57	0,59	0,14	0,55	0,92
Follicle TAS	r	0,01	0,01	0,04	0,20	0,42*
	p	0,94	0,95	0,82	0,24	0,01*
Follicle TOS	r	-0,14	-0,11	-0,03	0,19	-0,13
	p	0,39	0,50	0,98	0,27	0,43
Follicle OSİ	r	-0,16	-0,15	-0,02	-0,20	-0,20
	p	0,34	0,38	0,88	0,23	0,24

TAS: Total Antioxidant Status; TOS: Total Antioxidant Status; OSI: Oxidative Stress Index

The study divided the participants into those who became pregnant and those who did not, and their serum and FF levels of TAS, TOS, and OSI were analyzed (Table 6). The results indicated that the mean TAS values derived from serum were significantly higher in participants who achieved pregnancy than those who did not (p=0.04). However, no statistically significant differences were observed between the groups regarding other variables.

**Table 6:** TAS, TOS, and OSI values in participants who became pregnant and those who did not

	Pregnant (n=10)	Not pregnant (n=25)	p-value
Follicle TAS	1,71±0,34	1,80±0,38	0,86
Follicle TOS	9,81±2,50	8,23±4,56	0,09
Follicle OSİ	573,06±94,20	483,82±267,94	0,31
Serum TAS	1,48±0,64	1,12±0,31	0,04*
Serum TOS	20,91±9,45	21,13±11,75	0,48
Serum OSİ	2018,28±1122,72	1937,93±1303,66	0,83

TAS: Total Antioxidant Status; TOS: Total Antioxidant Status; OSI: Oxidative Stress Index

In the study, the FF and serum levels of TAS, TOS, and OSI were examined in participants who experienced neonatal death while in the hospital postpartum. However, it was determined that these levels were similar across the groups, and no significant differences were identified between them (Figure 1 and 2).

## Discussion

In contemporary times, the elevation of educational attainment has consequentially deferred the age at which individuals enter into matrimony, thereby advancing the age at which they procreate and altering the age distribution of infertility.^(14)^ Infertility is defined as the inability to conceive following one year of unprotected sexual intercourse. Its etiology may be attributable to factors about either the male or female partner.^(1)^ As the prevalence of infertility escalates, there is an increasing reliance on assisted reproductive technologies for treatment. Presently, the most prevalent method among these is the ICSI procedure.^(15)^

In this method, the follicle is cultivated until it attains a specific size. A single sperm is injected into the oocyte's cytoplasm. The developed embryo or embryos are transferred at the most opportune time. Post-treatment, numerous parameters significantly influence the achievement of a healthy pregnancy.^(16)^ Our study investigates the relationship between achieving a healthy pregnancy and TAS, TOS, and OSI values.

TAS, TOS, and OSI are critical parameters in evaluating oxidative stress and the antioxidant defense mechanism within biological systems. These metrics offer a comprehensive overview of the balance between oxidants, which are molecules capable of causing cellular damage through oxidation, and antioxidants, which are agents that counteract or neutralize this effect.^(6)^

TAS represents the cumulative effect of all antioxidants in plasma or serum, providing an integrated measure of the antioxidant capacity. It reflects the overall ability of the organism's defense system to counteract oxidative stress, encompassing both endogenous defenses and exogenous factors derived from diet and lifestyle.

TOS, on the other hand, quantifies the total level of oxidant molecules in the organism. It assesses the extent of oxidative damage potential, accounting for various reactive species such as reactive oxygen and reactive nitrogen. These species are typically byproducts of normal cellular metabolism but can accumulate to harmful levels under pathological conditions or environmental stress.^(17)^

The OSI is a calculated parameter that provides a ratio of TOS to TAS, offering a nuanced view of the oxidative balance. It is a significant indicator of oxidative stress, with higher values denoting a shift towards a pro-oxidant state. This imbalance between oxidants and antioxidants is implicated in the pathogenesis of numerous chronic diseases, including cardiovascular diseases, diabetes, neurodegenerative disorders, and cancer.^(18)^

Assessing TAS, TOS, and OSI furnishes crucial insights into an organism's oxidative stress status and antioxidative defense capacity, elucidating the intricate interplay between different molecular entities in the maintenance of cellular and systemic homeostasis. The overabundance of reactive oxygen species precipitates lipids, DNA, and protein degradation. This leads to mitochondrial and nuclear DNA damage and lipid peroxidation. Unsaturated fatty acids and other lipids undergo oxidation, transforming into peroxides. The resultant compounds disrupt cellular function by impairing cell membrane integrity and altering receptor activities.^(19)^

A study investigated the roles of FF and serum TAS, TOS, and OSI in the etiopathogenesis of unexplained infertility and ICSI-ET's success. The research was conducted with twenty male-factor unexplained infertility patients and an equal number of controls. Embryo quality and implantation, clinical pregnancy, and live birth rates were examined. It was found that the TOS and OSI values in the FF of patients with unexplained infertility were higher than those in the control group. In serum analysis, TOS and OSI were significantly higher than those of the control group. However, after age adjustment, only serum TOS levels were found to be positively associated with unexplained infertility. After age adjustment, the OSI value obtained from FF was negatively correlated with embryo quality in the unexplained infertility group. TAS, TOS, and OSI had no significant effect on implantation, clinical pregnancy, and live birth rates. The TOS value in serum may have a limited role in the etiopathogenesis of unexplained infertility, and the OSI value in FF could potentially reduce embryo quality in patients with unexplained infertility.^(20)^

Our findings indicate no significant differences between groups concerning TAS, TOS, and OSI. A positive and significant correlation was observed between embryo grade and the TAS values detected in serum and FF. Similarly, serum TAS levels exhibited a significant variance between participants who became pregnant and those who did not, with the pregnant participants displaying higher values. The outcomes of both studies corroborate the hypothesis that serum TAS levels influence embryo development.

In a study conducted by Ozturk et al., patients diagnosed with infertility were examined to assess the impact of TAS, TOS, and OSI levels in serum and FF on oocyte development, fertilization, embryogenesis, and clinical pregnancy outcomes during IVF treatments. The study included 100 patients undergoing IVF protocols. Blood samples were collected on the day of gonadotropin initiation, the day of embryo transfer, and the day of oocyte retrieval. FF was also collected during oocyte retrieval. TAS, TOS, and OSI levels in the serum and FF were compared between patients who achieved clinical pregnancy and those who did not. No significant differences were found in parameters such as age, duration of infertility, ovarian reserve, and the number of transferred embryos between pregnant and non-pregnant patients. Likewise, no differences were observed in the TAS, TOS, and OSI levels in the serum and FF. The research concludes that TAS, TOS, and OSI values in patients resorting to assisted reproductive techniques do not seem to predict clinical pregnancy outcomes effectively.^(21)^

The outcomes of this study resemble our results. Our research identified a correlation between embryo grade and the TAS values in serum and FF. Additionally, the serum TAS levels in pregnant participants were significantly elevated. The variations between the studies may be associated with the number of participants.

According to the study conducted by Singh et al.,^(22)^ patients with endometriosis and tubal infertility were compared in terms of oxidative stress markers. The results indicated that the TAS values in patients with endometriosis were higher compared to those with tubal infertility. However, when considering poor oocytes and embryo quality, a similar pattern in TAS values was not observed.^(22)^

This study's findings are parallel to our own, wherein a significant correlation between embryo grade and TAS values was identified. However, due to the discrepancy in statistical analyses, a robust comparison between the studies could not be established.

In the randomized controlled study conducted by Gong et al.,^(23)^ the effects of growth hormone on poor ovarian response in IVF patients were investigated. The study examined the mechanism by which growth hormone ameliorates oxidative stress. It was determined that in cases of poor ovarian response, the levels of TAS and OSI in the FF were significantly higher. At the same time, the TAS was significantly lower compared to the control group.^(23)^

Pregnancy-induced hypertension and intrauterine growth restriction are recognized as causative factors for perinatal morbidity and mortality. Research has concentrated on the role of oxidative stress as a pathophysiological mechanism in developing these pathologies. In this context, a study conducted by Zygula et al.^(24)^ compared the levels of oxidative stress between pregnant women with these conditions and those without complications. It was observed that in the group with intrauterine growth restriction, there was an increase in TAS both in saliva and plasma. In contrast, no increase was noted in other oxidative stress markers in the plasma.^(24)^

## Conclusion

In our study, it has been confirmed that serum TAS levels influence embryo development. A correlation has been identified between embryo grade and TAS values in both serum and follicular fluid (FF), with serum TAS levels being significantly elevated in pregnant participants.
